# Intraprostatic fiducial markers: a potential application for ultrasound-guided radiotherapy in prostate cancer

**DOI:** 10.3332/ecancer.2009.143

**Published:** 2009-05-12

**Authors:** A Vavassori, BA Jereczek-Fossa, D Zerini, L De Cicco, R Cambria, F Cattani, C Garibaldi, M Ciocca, R Orecchia

**Affiliations:** 1 Department of Radiation Oncology, European Institute of Oncology, 20141 Milan, Italy; 2 Department of Medical Physics, European Institute of Oncology, 20141 Milan, Italy; 3 University of Milan, European Institute of Oncology, 20141 Milan, Italy

## Abstract

We report on a two-phase test performed to assess the ability of the ultrasound-based B-mode acquisition and targeting (BAT) trans-abdominal system to identify non-dedicated fiducial markers implanted into the prostate gland for subsequent image-guided radiotherapy.

Although further investigation is warranted in order to identify the optimal echogenic marker and to define its potential use for image-guided radiotherapy in prostate cancer patients, we demonstrate the feasibility of the BAT system for the visualization of non-ultrasound-dedicated markers.

## Introduction

Dose escalation improves biochemical and clinical control in prostate cancer treated with external beam radiotherapy (EBRT), as demonstrated by several retrospective and prospective randomized studies [1–5]. Whenever a dose of >70 Gy is administered, the irradiated volume should be reduced in order to avoid excessive damage to the surrounding normal tissue. In clinical practice, such volume reduction has become feasible with the introduction of image-guided radiotherapy (IGRT). Various systems are routinely used in many departments for daily localization of the prostate during radiotherapy, i.e. implanted fiducial markers with x-ray, on-board computed tomography (CT) or dedicated ultrasound (US) IGRT systems. Among these methods, the US system is probably the fastest. Several authors report that the fiducial marker-based IGRT is the most reliable, although it does not allow for soft tissue (prostate, seminal vesicles, rectum, urinary bladder) imaging [6].

In June 2005, the B-mode acquisition and targeting (BAT) transabdominal US System (Nomos, USA) was installed in our department. It is a computer-assisted cart-based system located in the treatment room enabling daily patient setup.

In a previous study [7], we evaluated the feasibility and accuracy of BAT-based prostate localization. Ten patients with localized prostatic adenocarcinoma were treated with three-dimensional conformal radiotherapy (3D-CRT) to the dose of 72 Gy/30 fractions prescribed in the ICRU point (International Commission of Radiation Units). Daily US-based IGRT was compared to the electronic portal imaging (EPI) and CT-based alignment. We demonstrated that the BAT system ensures that the relative positions of the isocentre remain the same during treatment and as outlined in the treatment plan, even if the reliability of alignment is patient dependent. The average BAT-determined misalignments were small, confirming the prevalence of random errors in 3D-CRT. The limitations of the BAT system include inaccuracy of comparing images acquired by two different modalities (CT for planning and daily US images for setup), possible prostate displacement induced by US-probe pressure and operator dependence [8]. According to the literature, an average probe displacement of 12 mm results in an average prostate displacement of 3 mm [9,10]. Prostate movement due to probe pressure is mostly in the anteroposterior (AP) direction, but systematic shifts have been observed also in the craniocaudal direction [11,12].

An unpublished study on a series of 25 patients affected by localized prostate cancer treated at our department with hypo-fractionated 3D-CRT and daily BAT-based prostate localization showed a mean inter-patient systematic displacement of the prostate of 2.4 mm in the AP direction, probably related to the different US probe pressures used.

The purpose of this study was to test the potential role of two different commercially available fiducial markers in the US-based IGRT for prostate cancer. In the future, the introduction of fiducial markers for US-based IGRT might reduce both operator-dependent variations and potential influence of unfavourable patient anatomy, such as obesity or a small prostate located behind the pubic symphysis (patient dependence). Moreover, errors introduced during the learning curve for inexperienced US operators could be reduced.

## Materials and methods

We tested the ability of the BAT system to visualize a set of standard gold fiducial markers (VisiCoil, RadioMed Corporation, USA; 0.75 mm in diameter and 10 mm in length) already used in our department for ExacTrac prostate cancer IGRT. In ExacTrac patients, the two VisiCoil fiducial markers are introduced into the prostate seven days before the simulation. For the purpose of the present study, we performed the BAT procedure in one patient enrolled in our ExacTrac x-ray hypofractionated protocol (2.7 Gy fraction; five fractions/week; 26 fractions, total dose of 70.2 Gy).

The second part of our study involved the evaluation, in a low-risk prostate cancer patient, selected for permanent seed implant brachytherapy, of the ability of the BAT system to visualize a different type of commercially available marker (CyberMark™ Fiducial Marker, Civco, USA, 1 mm in diameter and 5 mm in length).

A dedicated informed consent was obtained. The implantation of the fiducial markers, near the left and right base positions, was performed using the same template-based brachytherapy technique.

Under epidural anaesthesia, the bladder was catheterized and filled with 200 cc of saline solution. A well-trained operator performed a preliminary transabdominal US examination, using the BAT system, to check prostate visibility.

Each marker was implanted via a transperineal percutaneous approach, using a dedicated preloaded needle under real-time transrectal US guidance.

At the end of the procedure, the same operator performed a new transabdominal US evaluation with the BAT system to visualize the markers inside the prostate and thereafter the planned brachytherapy procedure was started.

## Results

These preliminary tests demonstrated the ability of the BAT system to visualize non-US-dedicated markers.

**Figure 1:**
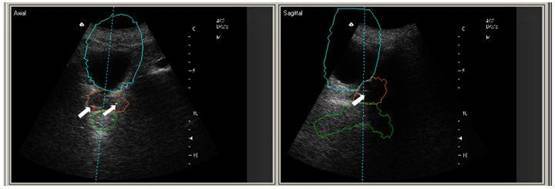
US-images and simulation-CT contours of the bladder, prostate and rectum in the IGRT patient with VisiCoil markers implanted (white arrows).

The alignment based on the fusion of the fiducial marker images coming from the simulation-CT and BAT procedure was feasible. The marker-based shift was equal to the soft-tissue imaging-based shift. The quality of the marker US image was satisfactory (Figure 1).

**Figure 2:**
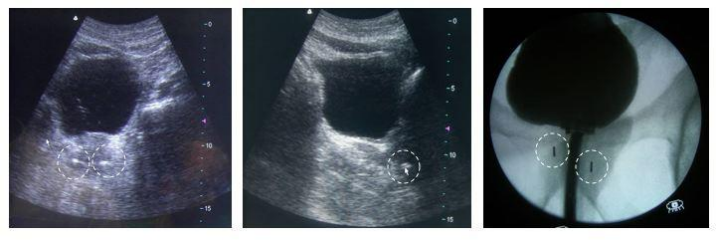
US and x-ray images of the CyberMark™ markers (dotted circles) in the brachytherapy patient.

The time needed for implanting the markers in the brachytherapy patient was about 20 minutes and did not affect the accurate completion of the planned permanent seed implant (Figure 2).

The patient experienced no complications or discomfort after the two procedures.

## Discussion

We have demonstrated the capacity of the BAT system to visualize different types of standard and commercially available non-US-dedicated fiducial markers.

The marker implantation is an invasive procedure with a potential for discomfort, possible bleeding, infection and prostate deformation [13]. However, if it proves applicable in clinical practice for US-based IGRT, as has been already demonstrated for cone-beam CT IGRT, it could reduce the subjectivity involved in interpreting the soft tissue image. In this way, the need for additional radiation doses from MV or kV radiography could be avoided. [14].

Moreover, the cost of US-based IGRT, even when fiducials are used, should be competitive with the CT or x-ray-based IGRT procedures.

## Conclusions

Although the feasibility of using the BAT system with fiducial markers has been clearly demonstrated, new echogenic fiducials with different technical design should be tested to in order to facilitate the transabdominal US image evaluation [15].

Their construction material (gold, titanium, silver, carbon, etc.) should be carefully chosen to increase the proportion of US signal reflected depending on their different acoustic impedances. We believe that the results of this preliminary investigation warrant a more complete clinical study of the practical applicability of radiopaque echogenic markers in patients selected for daily BAT-based IGRT. Such a procedure might reduce inter-fraction and inter-observer discrepancies. Future studies, including 3-D-US technology, are warranted in order to increase the accuracy of US-based IGRT for prostate cancer.
